# Spontaneous Cecal Perforation During Pregnancy

**DOI:** 10.7759/cureus.55862

**Published:** 2024-03-09

**Authors:** Abdalhai Alshoubi, Melissa Kim, William Hauter, Waqqas Khan, Harsh Shah

**Affiliations:** 1 Anesthesiology and Critical Care, OSF St. Francis Hospital, Peoria, USA; 2 Anesthesiology and Critical Care, University of Illinois College of Medicine Peoria, Peoria, USA; 3 Anesthesiology, OSF St. Francis Hospital, Peoria, USA

**Keywords:** primary suture, pregnancy, idiopathic, spontaneous perforation of the colon, cecal perforation

## Abstract

Spontaneous perforation of the colon is a rare disease defined as sudden perforation of a healthy colon without evidence of trauma or disease. These perforations are typically classified as either stercoral or idiopathic. Cecal perforation during pregnancy is an uncommon and potentially life-threatening condition requiring prompt recognition and surgical intervention.

We present a case of a 33-year-old woman at 29 weeks and three days gestation presenting with spontaneous cecal perforation. She presented to the emergency department with diffuse abdominal pain and distention lasting for three days, associated with nausea and vomiting. Following evaluation, she was diagnosed with diffuse peritonitis. The diagnosis of this condition relies on both the clinical presentation and the utilization of radiographic imaging.

The patient underwent an emergent explorative laparotomy with prompt surgical intervention to repair the 1.2 x 0.8 cm perforation found on her distended cecum. The surgical repair consisted of the excision of the edges and the primary suture of the perforation with an omental patch. Her post-procedure course was uneventful, and she later delivered a healthy baby at full term. This case highlights the importance of considering uncommon causes of acute abdominal pain in pregnant women to ensure timely diagnosis and management.

## Introduction

Spontaneous cecal perforation is a rare surgical emergency associated with high morbidity and mortality rates. The majority of spontaneous colon perforation happens in the sigmoid colon. It is defined as the sudden perforation of normal colon mucosa in the absence of injury or malignancy, and very few cases have been reported [1]. Although various etiologies may underlie the pathophysiology, spontaneous perforation in the absence of predisposing factors is exceedingly rare, particularly in pregnant women. The clinical presentation of patients can vary, with the majority of cases presenting with diffuse peritonitis.

The diagnosis can be challenging, especially for pregnant women, due to the unique anatomical and physiological changes of pregnancy. Due to its rarity, spontaneous cecal perforation is typically diagnosed intraoperatively and is often linked with high mortality rates. The surgical repair of cecal perforation in a pregnant patient includes primary repair with an omental patch or right hemicolectomy. Here, we present a case of spontaneous cecal perforation in a 33-year-old pregnant woman who underwent primary repair with an omental patch.

## Case presentation

A 33-year-old woman, gravida 3, para 2, presented at 29 weeks and three days gestation. The patient's height was 156 cm, and her weight was 101 kg, with a body mass index of 41.5 kg/m2. She was obese but otherwise healthy, with no history of chronic constipation or hematochezia. She has not undergone any surgeries and has previously had uneventful vaginal deliveries. Her antenatal care has all been normal, and she has reported normal fetal movements throughout her pregnancy. She has not experienced kidney stones or any urinary symptoms. She presented to the emergency department complaining of generalized abdominal pain associated with nausea, vomiting, and fever. The patient hadn't had a bowel movement for three days before admission. She typically has two bowel movements per week.

Upon examination, her vital signs showed a blood pressure of 112/65 mmHg, a pulse of 121 beats/minute, a respiratory rate of 23 breaths/minute, an oxygen saturation of 96% on room air, and a body temperature of 38.2°C. Her abdomen was rigid and tender throughout, with evidence of generalized rebound tenderness. Bowel sounds were diminished, and a cervical examination revealed a long and closed cervix. Heart and lung examinations were unremarkable. Fetal ultrasonography showed a normal intrauterine fetus at 30 weeks of gestation. Laboratory analysis revealed leukocytosis with neutrophilia (Table 1). The electrolyte profile and lactate levels were normal. Urinalysis was unremarkable, and no X-ray, magnetic resonance imaging (MRI), or computed tomography (CT) scan was performed.

**Table 1:** Serum laboratory results on the first and last days of admission WBC: white blood cells; RBC: red blood cells; BUN: blood urea nitrogen; AST: aspartate transferase; ALT: alanine transaminase; INR: international normalized ratio; PT: prothrombin time; PTT: partial thromboplastin time; uL: microliter; g/dL: grams per deciliter; fL: femtoliters; mmol/L: milimoles per liter; mg/dL: milligrams per deciliter; U/L: units per liter.

Parameter	Reference range and units	First day of admission	Last day of admission
WBC count	4-10 10^3^/uL	16.7	7.3
RBC count	4.3-5.9 10^6^/uL	3.83	3.14
Hemoglobin	14-18 g/dL	10.8	9.1
Hematocrit	39-49%	31.4	27.9
Mean corpuscular volume	80-99 fL	76.8	87.4
Red cell distribution width	11.4-14.6%	19.5	21.1
Platelet count	150-400 10^3^/uL	240	261
Lymphocytes	16-45%	8	23
Neutrophils relative percent	42-75%	87	61
Monocytes	2-12%	6	9
Eosinophils	0-5%	1	0
Basophils	0-2%	0.1	1
Sodium	135-145 mmol/L	141	138
Potassium	3.5-5.1 mmol/L	4.1	4.6
Chloride	98-107 mmol/L	100	101
Carbon dioxide	21-32 mmol/L	24	23
Glucose	74-106 mg/dL	126	120
BUN	7-18 mg/dL	8	11
Creatinine	0.70-1.30 mg/dL	1.01	1.0
Calcium	8.5-10.1 mg/dL	9.6	9.1
Phosphorus	2.3-4.7 mg/dL	2.1	2.4
AST	15-37 U/L	23	21
ALT	16-61 U/L	19	26
Protein, total	6.4-8.2 gm/dL	6.1	5.1
Albumin	3.4-5 gm/dL	2.8	2.6
Alkaline phosphatase	40-150 U/L	43	64
Bilirubin, total	0.3-1 mg/dL	0.9	1.1
INR	1	1	1.1
PT	9.4-12.5 seconds	12	13
PTT	25.1-36.5 seconds	32	31
Lactic acid	0.5-2 mmol/L	1.2	1.1

The patient was diagnosed with diffuse peritonitis and underwent an emergent exploratory laparotomy. Operative findings showed an anterior cecal perforation measuring 1.2 x 0.8 cm. Peritoneal cavity irrigation with saline was performed, the perforation was repaired by a primary suture with an omental patch after trimming the edges, and an abdominal drain was placed. The rest of the colon appeared normal, with no masses, inflammation, or obstruction. The appendix and ovaries were normal. The cause was considered idiopathic. The postoperative course was uneventful, and the patient was discharged home in stable condition on postoperative day four. She later had an uneventful, normal vaginal delivery at 38 weeks of gestation.

## Discussion

Cecal perforation during pregnancy is a rare but potentially catastrophic event, with very few reported cases in the literature. The etiology of spontaneous cecal perforation remains unclear. Most non-traumatic causes of cecal perforation can be divided into stercoral and idiopathic causes. The first occurs due to increased compression and subsequent ischemia and necrosis of the colonic wall by solid fecal matter in the setting of chronic constipation. This is frequently seen in elderly patients with a history of constipation with a mean age of onset greater than 65 [1]. The proposed diagnostic criteria for feculent colonic perforation are as follows: a rounded shape exceeding 1 cm in diameter; fecaloma present diffusely throughout the colon and in the abdominal cavity through the perforation; ischemia and necrosis of the colonic mucosa leading to a chronic inflammatory response visible around the perforation site microscopically; and exclusion of injury, obstructions, tumors, or diverticulosis [2]. In contrast, idiopathic perforations appear as linear tears with clear edges and normal-appearing mucosa both macroscopically and microscopically [3]. Predisposing factors include the hypoperfusion of colonic tissue combined with the asymmetrical distribution of intraluminal pressure, leading to hyperdilation and constitutional weakness of the bowel wall. Spontaneous cases of perforation have also been reported among young children as a manifestation of connective tissue disorders such as Ehlers-Danlos Syndrome Type IV or Marfan Syndrome [4]. Among reported cases of cecal perforations, there appears to be a bimodal distribution of age, but our case demonstrates that it is not limited to the elderly or infants.

Certain conditions, like hypothyroidism and intestinal hypomobility, that are encountered during pregnancy should be explored as potential risk factors for spontaneous cecal perforations in this subpopulation. Hormonal changes during pregnancy can decrease intestinal transit and lead to bowel dilation, a predisposing factor for ischemia and perforation. The cecum’s wide diameter makes it particularly vulnerable, as it can easily distend with minimal intraluminal pressure according to Laplace’s Law and exceed the capillary perfusion pressure in the bowel walls [5]. Other rare causes include perforated right diverticulitis, tumors or foreign bodies, infectious etiologies like tuberculosis, iatrogenic endoscopic procedures, and C-sections [6]. Multiple modalities can be used to confirm a diagnosis along with the clinical presentation. These modalities include ultrasound, MRI, and CT scans. The National Council of Radiation Protection and Measurements, the American Council of Obstetricians and Gynecologists, and the American College of Radiology unanimously opined that the risk of ionizing radiation-induced fetal harm is considered negligible at 50 milliGray (mGy) or less, and the risk of malformations increases significantly only at doses above 150 mGy. The regular radiation dose from abdominal CT is generally less than 15 mGy.

Two main surgical approaches are considered for the repair of a cecal perforation: primary suture or right hemicolectomy. In the absence of severe infection and well-controlled hemostasis, primary suture repair and omental patching are much less invasive procedures [7]. In cases of severe inflammation, neoplasm, or indications of necrosis, a right hemicolectomy is recommended but is also associated with higher risks of morbidity and mortality [8]. Potential advantages to primary suture repair are a shortened postoperative stay, less blood loss, and lower risks for anastomosis breakdown [9,10]. Post-surgical outcome depends on time of onset, degree of perforation and peritoneal contamination, and the vital status of the patient. In our case, prompt recognition and management led to favorable outcomes for both the mother and fetus.

## Conclusions

Spontaneous cecal perforation during pregnancy is a rare but serious condition that requires prompt surgical management. Clinicians should maintain a high index of suspicion for this uncommon pathology in pregnant women presenting with acute abdominal pain, as timely intervention can significantly impact maternal and fetal outcomes. Irrespective of the surgical approach, early detection and prompt surgical intervention are the primary strategies linked to enhanced outcomes. Further research is warranted to better understand the risk factors and optimal management strategies for this rare condition.
